# Nutritional knowledge of youth academy athletes

**DOI:** 10.1186/s40795-020-00360-9

**Published:** 2020-08-18

**Authors:** Stephen P. Bird, Benjamin D. Rushton

**Affiliations:** 1 School of Health and Wellbeing (Sport and Exercise Science), University of Southern Queensland, Ipswich, QLD Australia; 2 School of Exercise Science, Sport and Health, Charles Sturt University, Bathurst, NSW Australia; 3 School of Exercise and Nutrition Sciences, Faculty of Health, Deakin University, Melbourne, VIC Australia

**Keywords:** Nutrition knowledge, Supplements, Youth athletes

## Abstract

**Background:**

Youth athletes are under increasing pressures to excel in their chosen sport and many turn to nutritional supplements in order to enhance sports performance. However, athletes may obtain their nutritional information via illegitimate sources such as the internet, media, and other athletes, representing miscommunication between sound scientific information and anecdotal experiences. The objective of this investigation was to examine nutrition knowledge of elite youth athletes from a non-residential regional academy of sport.

**Methods:**

A previously validated two-part nutrition knowledge questionnaire (NKQ) was administered to 101 (37 male and 64 female) non-residential regional Academy of Sport elite youth athletes at an annual training camp. Part 1 of the NKQ presented demographic questions. Part 2 presented 90 sports nutrition knowledge questions in seven knowledge subcategories (1) Nutrients; (2) Dietary reference intakes (DRI); (3) Fluids/Hydration; (4) Recovery; (5) Weight gain; (6) Weight loss; and (7) Supplements.

**Results:**

The mean NKQ score of all athletes was 43.8% (± 11.4). No gender differences observed between nutritional knowledge total scores, however female athletes recorded more ‘correct’ responses than males (*p* = 0.02) in the Nutrients subcategory. Majority of athletes had difficulty identifying correct DRI with this subcategory featuring the lowest percentage of ‘correct’ to ‘incorrect’ responses (27.1% ± 2.3; *p* = 0.02). Supplements subcategory displayed much uncertainty with significantly more ‘unsure’ than ‘incorrect’ responses (42.4% ± 20.3; *p* < 0.05).

**Conclusions:**

In agreement with previous research, results of the current study indicate that elite youth athletes lack fundamental nutritional knowledge, specifically related to DRI and supplementation. These data provide further support of current recommendations that Academy of Sport youth athletes may benefit from integrated nutrition education conducted by qualified nutrition professionals.

## Background

Adolescence is defined by the World Health Organization [1] as a period of significant growth and maturation occurring following childhood and prior to adulthood, with adolescents considered as those between the ages of 10 to 19. It is well established that this period of significant growth and physical development can be negatively affected by malnutrition [2, 3], as such proper nutrition is of significant importance especially in the competitive adolescent athlete with the added demands of training and competition [4, 5]. In recent times there has been a rise in youth sports participation [6], however youth athletes may be increasingly pressured to excel in their sport, whether through parents, sporting coaches or intrinsic pressures imposed by the athlete [7, 8]. In an attempt to gain a competitive edge and improve performance many youth athletes turn to nutritional supplements [9–12]. However, research indicates that youth athletes possess limited nutritional knowledge [13, 14], with much of their nutritional information obtained via illegitimate sources such as the coach, teacher, other athletes, internet, and social media [15]. Potentially, this may represent a miscommunication from non-qualified individuals providing nutritional advice to athletes that leads to nutritional principles being misunderstood and/or incorrectly applied [16].

Research suggests that collegiate athletes with greater understanding of sound nutritional principles are more likely to apply this knowledge and display positive nutritional behaviors [17]. This is an extremely important consideration, especially for athletes competing in weight-restricted or aesthetic sports wherein youth athletes may feel pressured to restrict caloric intake [18, 19]. Unknowingly, not only may this compromise the athlete’s physical development, such nutritional habits may predispose psychological behaviors towards disordered eating traits. Smith-Rockwell et al. [20] revealed that in Division I collegiate coaches/trainers 35% reported at least one perceived eating disorder case per year, while 10% reported more than three cases per year. Although many coaches/trainers referred athletes with eating disorder symptoms to physicians, almost one third dealt with these cases themselves. Interestingly, only 30% of collegiate athletes had access to a sports dietitian, and the same percentage reported utilizing dietitians for nutritional advice [20]. Collectively, this would suggest that reliable sources of nutrition information and education, as well as appropriate nutrition services including counseling are required for collegiate athletes.

Within elite sporting environments there appears to be an increasing trend of coaches and athletic trainers providing nutritional advice [20–22], and this seems to be common practice especially for strength and conditioning coaches [20, 23]. A survey of elite rugby union coaches [16] reported that despite responding correctly to only 55% of all questions on the Nutrition Knowledge Questionnaire (NKQ) [24], 83% provided nutritional advice to their athletes. Studies assessing coach/athlete nutritional knowledge reported a 67% correct response rate [20]. Further research solely in athletic populations (age range: 18–37 yrs) using the same or modified iterations of the NKQ, found nutritional knowledge in elite Australian rules footballers (61%) [25], professional (54%) and semi-professional soccer players (56%) [26], is well below the adequate sports nutrition knowledge overall score of 75% as proposed by Torres-McGehee et al. [23] This would suggest that both athletes and coaches lack sports nutrition knowledge. Although concerning, the apparent lack of nutritional services available to athletes may be responsible, in part, for necessitating this trend.

While nutritional knowledge and source of nutritional information has been extensively reported in collegiate athletes and coaches/trainers [9, 20, 25–30], to date, there are no published data on youth athletes from Regional Academy of Sport programs. Recently, Spronk et al. [14] reported that a substantial proportion of what were predominantly youth athletes (16–18 yrs) failed to meet basic dietary recommendations, especially dairy intake. Therefore, it is unclear whether youth athletes have an appropriate level of general nutrition knowledge and understanding of nutrition principles, as much of the literature has focused upon collegiate and semi- or professional athletes [13, 16, 20, 25–28]. Given the potential for a lack of exposure to high quality nutrition-related education, nutrition counselling, and qualified sports nutrition professionals youth athletes may be ill-equipped to make accurate decisions regarding their nutritional requirements, and this in turn may negatively impact their health status, physiological development and/or sports performance.

The purpose of the current study was to examine and describe general nutrition knowledge and nutrition-related practices of youth athletes from a non-residential Regional Academy of Sport in NSW Australia. Specifically, this study shall quantify nutritional knowledge in youth athletes across a range of academy sports, as well as identifying athletes’ primary sources of nutritional information. It was hypothesised that regional youth academy athletes would display limited nutrition knowledge, and this would be related to their primary source of nutritional information.

## Methods

### Participants

A total of 101 elite youth athletes (37 males, 64 females; state and national competition level; 15.3 ± 1.4 years) who were scholarship holders from a non-residential regional Academy of Sport (Western Region Academy of Sport, WRAS) in Australia were invited to participate in this study. Athletes were representative of eight WRAS sports (Netball, Officiating, Basketball, Softball, Hockey, Tennis, Lawn bowls, and Triathlon). Descriptive data is presented in Table 1 (*n* = 101). After a full explanation of all procedures and possible risks of the investigation, written informed consent was obtained (for participants < 18 years, a legal guardian also provided written consent). All experimentation was approved by the Charles Sturt University Ethics in Human Research Committee.

**Table 1:** Participant characteristics

Variable	Youth Athletes (***n*** = 101)
*Age* (yrs)	15.3 ± 1.4 yrs
*Gender*	
Male	37
Female	64
*High School Level*	
Year 7 (13 yrs)	11
Year 8 (14 yrs)	19
Year 9 (15 yrs)	25
Year 10 (16 yrs)	21
Year 11 (17 yrs)	21
Year 12 (18 yrs)	4
*Years competing at this level*	
< 1 yr	26
2–4 yrs	63
5–7 yrs	10
8 + yrs.	2
*Training hours per week*	
4–6 h	40
7–10 h	42
11–14 h	15
15 + hrs	4

### Nutrition knowledge questionnaire (NKQ)

Nutritional knowledge was measured using a previously validated Nutrition Knowledge Questionnaire (NKQ) [16, 24]. Consultation with an expert panel consisting of a Registered Nutritionist; Sports Dietitian; two Academy Head Coaches; and an Academy Strength and Conditioning coach, was held. Each question was read out loud by the lead researcher and critiqued by the expert panel in a group discussion for comprehension, relevance and accuracy. If required, the wording was modified slightly to engage the target demographic of this survey (i.e., high school student-athletes; male and female; aged 13 to 18 years). The NKQ consisted of two distinct sections; Section 1 presented demographic questions including age, level of competition, training load expressed as hours of training per week, education level and primary source of nutrition information. Section 2 presented 90 sports nutrition knowledge questions in seven subcategories.

1. Nutrients
2. Dietary reference intake (DRI)
3. Fluids/Hydration
4. Recovery
5. Weight gain
6. Weight loss
7. Supplements

Each question had a potential answer of “yes”, “no” or “unsure”, with a correct response was coded as + 1 whilst incorrect or unsure responses were coded as 0. The rationale for the inclusion of an unsure response is to deter subjects from guessing responses in the NKQ as suggested by Zinn et al. [16] All athletes attended a nutrition research session in the same room at the annual training camp. The NKQ was distributed to athletes in hard copy format with the lead researcher providing specific instructions on how to complete the NKQ, which took taking approximately 45 min to complete under the supervision of the researchers.

### Statistical analysis

Descriptive statistics were used to analyze the demographic information. Nutrition knowledge sub-scores for each section and an overall nutrition knowledge total score was calculated. Percentage of ‘correct’, ‘unsure’, and ‘incorrect’ responses to each individual item was analyzed. Independent t-tests were used to show mean correct, incorrect, and unsure score differences between the two groups (male and female athletes). All data were coded, entered numerically and analyzed using SPSS version 21.0 (SPSS Inc., Chicago, IL, USA, 2012) with significance set at *p* < .05. Data are presented as percentages, means, and standard deviations.

## Results

### Nutritional knowledge

**Fig. 1:**
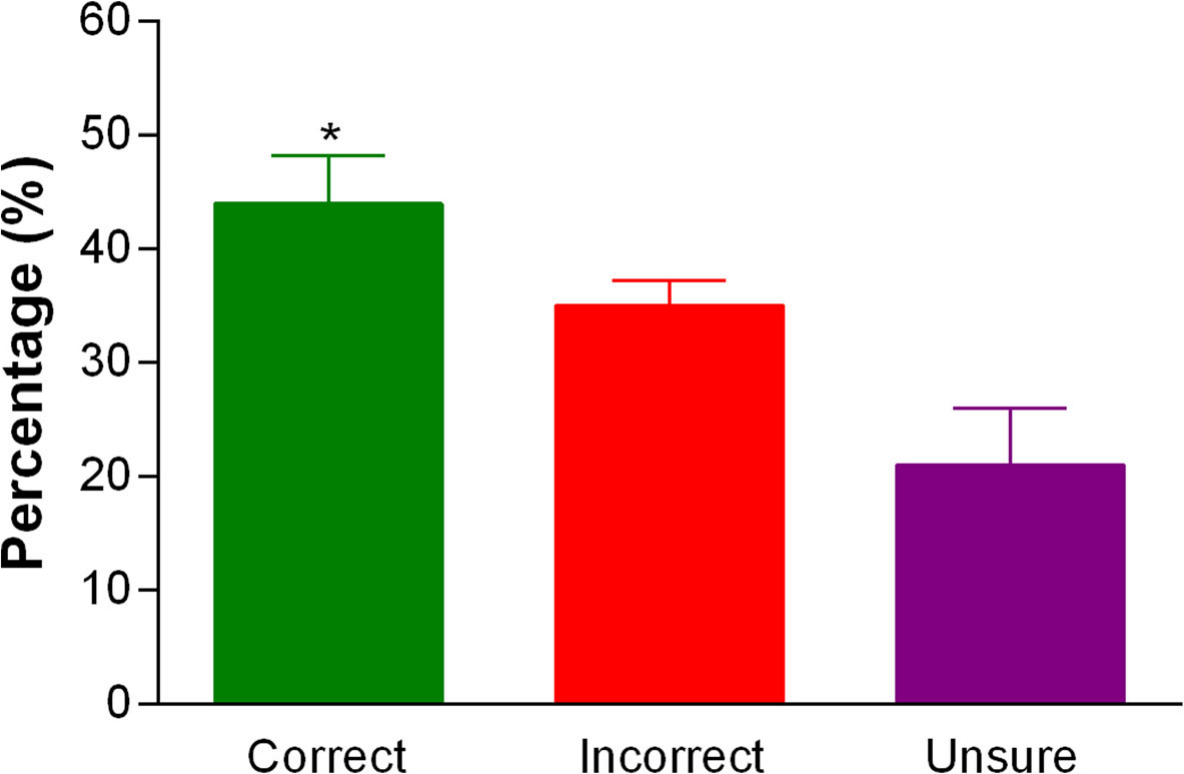
Mean percentage score of correct, incorrect and unsure total scores obtained by subjects on the nutritional knowledge questionnaire. * Significantly greater than ‘unsure’ and ‘incorrect’

Figure 1 presents the mean percentage of ‘correct’, ‘incorrect’, and ‘unsure’ total scores obtained by youth athletes on the NKQ. The mean overall score was 43.8 ± 11.4% (pooled data) with significantly more (*p* < 0.01) ‘correct’ responses compared to ‘unsure’ responses. No significant gender differences were observed between nutritional knowledge total scores, however subcategory differences were evident.

### Subcategory knowledge

**Fig. 2:**
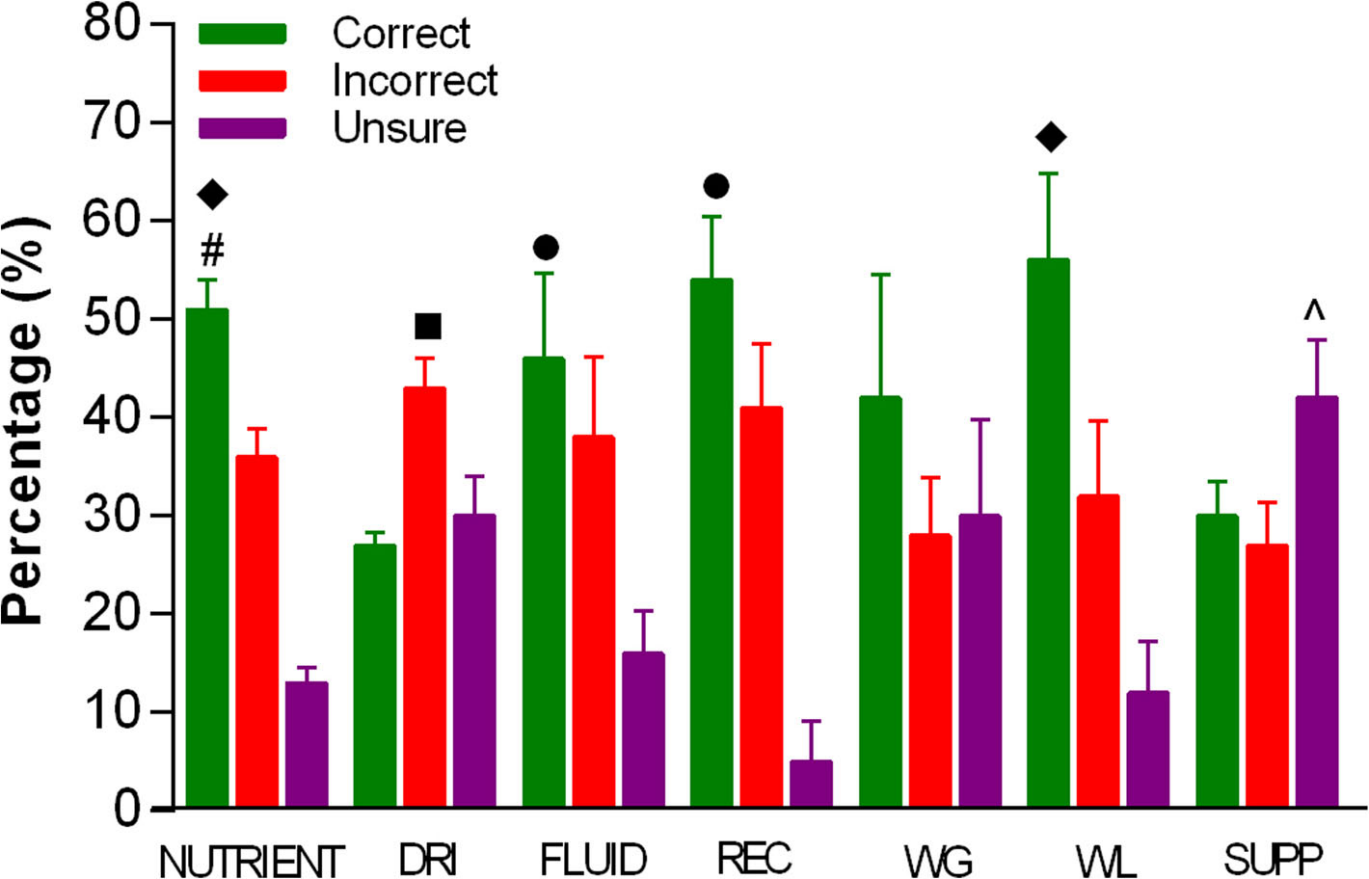
Subcategory knowledge mean percentage score of correct, incorrect and unsure totals scores across the seven subcategories. ◆ Significantly greater than ‘incorrect’ and ‘unsure’; ■ Significantly greater than ‘correct’; ● Significantly greater than ‘unsure’; ^ Significantly greater than ‘incorrect’; # Significantly more ‘correct’ responses for females than males. Abbreviations: DRI = Dietary reference intakes, REC = Recovery, WG = Weight gain, WL = Weight loss, SUPP = Supplements

Figure 2 displays the subcategory knowledge mean percentage of ‘correct’, ‘incorrect’, and ‘unsure’ total scores obtained by youth athletes on the NKQ.

#### Nutrients

Subjects correctly answered 51% of questions within this section. There was a greater number of ‘correct’ responses in this subcategory compared with ‘unsure’ (*p* < 0.01)*.* Interestingly, 72% of subjects incorrectly stated ‘avocados were low in fat’, while 51% incorrectly identified ‘chicken as a high carbohydrate food’. An independent t-test showed that females obtained more ‘correct’ responses than males (*p* = 0.02).

#### Dietary reference intakes

This subcategory was the most poorly answered of all the subcategories and featured the lowest percentage of ‘correct’ to ‘incorrect’ responses (27% vs. 43%, respectively; p = 0.02). Notably, 37 and 38% of athletes responded ‘unsure’ and ‘incorrect’, respectively, to the protein recommendations for youth athletes. Additionally, carbohydrate recommendations were incorrectly identified by 45% of athletes.

#### Fluids/hydration

Less than half (46%) of the fluid/hydration questions were answered correctly. Regarding fluid ingestion, 72% of respondents were unable to correctly identify the fluid requirements for an intense two-hour training session and only 9% correctly identified the carbohydrate content range of a standard sports drink.

#### Recovery

Only half (54%) of the recovery questions were answered correctly. Regarding carbohydrates and recovery, not only was the definition of Glycemic Index a source of confusion with 45% responding ‘unsure’ but 64% of athletes incorrectly identified green salad as containing more carbohydrates than soft drink.

#### Weight gain and weight loss

A substantial variation in mean correct percentage scores within this subcategory was evident with ‘correct’ responses ranging from 3 to 78%. More than half of the athletes (54%) reported that consuming protein powder is essential to increase muscle mass, while 67% were ‘unsure’ what type of protein was most suitable for gaining muscle mass. The weight loss section had significantly more (56%; *p* < 0.05) ‘correct’ responses compared to ‘incorrect’ and ‘unsure’.

#### Supplements

**Fig. 3:**
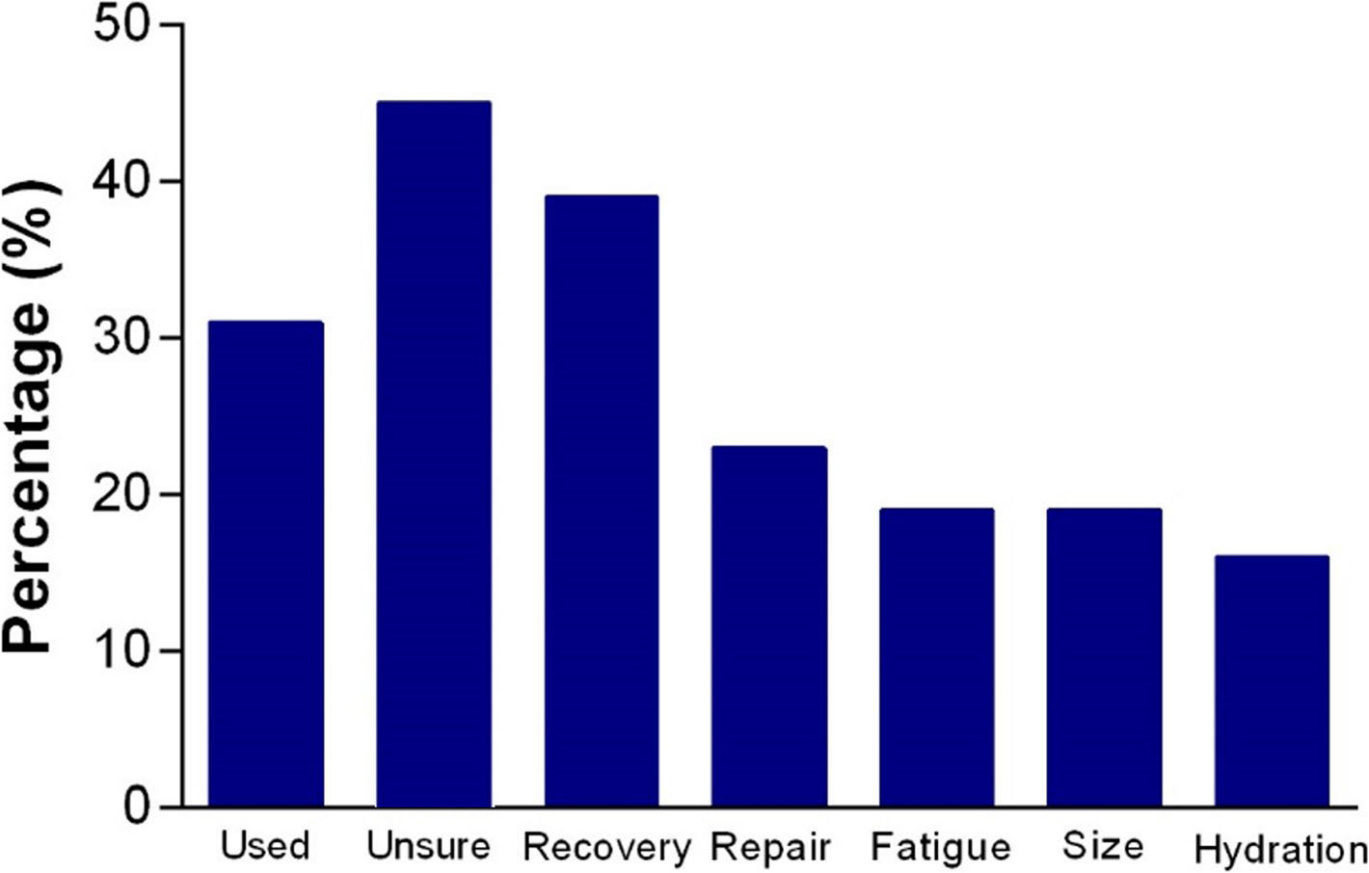
Protein supplementation. Percentage of youth athletes using a protein supplement in the last 12 months and the reason provided for usage

Collectively, the responses for this subcategory displayed much uncertainty with significantly more ‘unsure’ responses to these questions than ‘incorrect’ responses (42%; p < 0.05). One third of youth athletes reported protein supplement use over the previous 12 months (Fig. 3); however, athletes lacked knowledge as to why you would take this supplement with 45% of athletes responding ‘unsure’. Further questions pertaining to creatine supplementation and hydroxy-methyl butyrate (HMB) resulted in similar ‘unsure’ responses of 63 and 70%, respectively. Overall, there was a greater number of ‘unsure’ responses in this subcategory compared with any other subcategory (*p* = 0.02).

### Source of nutritional information

**Fig. 4:**
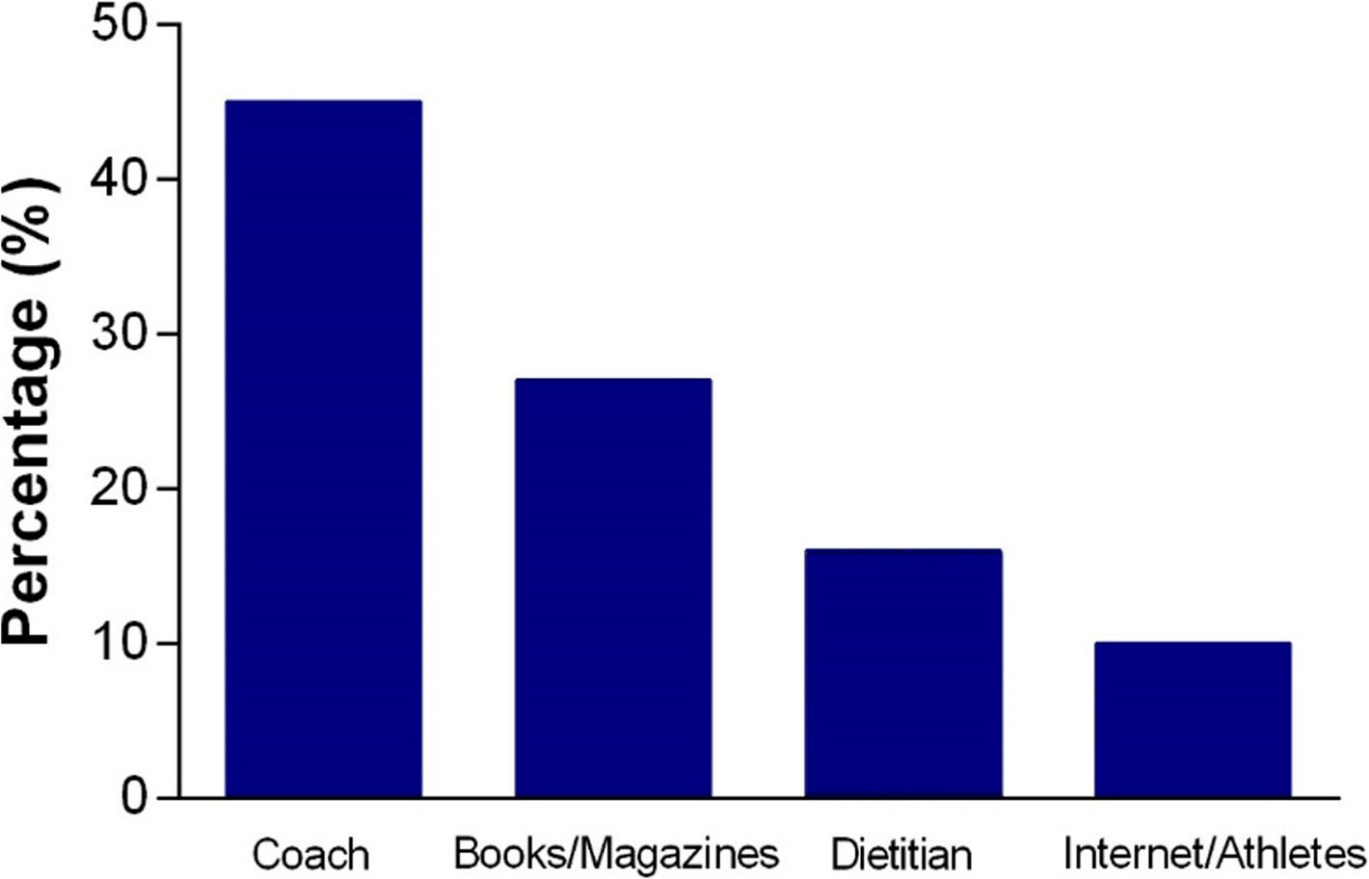
Source of nutrition information. Mean percentage score of where youth athletes source nutrition information

Figure 4 presents the reported sources of nutritional information of youth athletes. Notably, 45% of athletes reported that the coach was the primary source of nutrition information, followed by books/magazines (27%). Dietitians were only reported for 16% of athletes with minor sources of nutritional information including the internet and other athletes (10%).

## Discussion

Results from the current study indicate that youth athletes lack nutritional knowledge in multiple areas, with only two subcategories recording mean ‘correct’ responses above 50%. Athletes’ nutritional knowledge were particularly lacking pertaining to the areas of nutrient recommendations and supplementation, indicating the need for further nutrition education. This may predispose youth athletes to make inadequate nutrition and/or supplementation choices based on misconceptions and/or misinformation that may negatively impact growth, physical development and performance [4].

The results of current study further support recommendations for nutrition education programs for both youth athletes [31] and coaches [23], as Little et al. [31] has shown that as little as five nutrition education sessions can be effective in improving nutrition and supplement knowledge in youth athletes from low-income communities. This may be reflective of the youth athletes in the current study, with many of the geographical locations (inland regional and rural towns) recognized as low socioeconomic communities.

Our study revealed that the coach represented the main source of nutritional information for athletes (45%), whilst dietitians represented only 16%. Previous research has demonstrated that professional coaches recorded mean nutritional knowledge scores ranging from 48 to 67% [16, 20], as such it may be fair to assume that coaches of youth athletes may likely have comparable or lower levels of nutrition knowledge. However, this was not measured in the current study. The low incidence of dietitians being sourced to provide nutritional advice is of concern, however these data are similar to other reports [32]. Despite youth athletes in the current study being classified as ‘elite’, with competition level ranging from state, national and international, the rural location of the academy and the added expense of dietetic consultations likely presents barriers for athletes from regional and rural areas to source expert services. Smith-Rockwell et al. [20] reported that strength and conditioning coaches/trainers with access to a dietitian made full use of these services. Therefore, increased accessibility may result in greater usage of qualified sports nutrition professionals as the primary source of nutrition information and education. With greater access to nutrition support services, one could expect that athletes would exhibit higher nutritional knowledge [14, 25, 26].

Conversely, Burns and colleagues [32] found that even with access to an available dietitian, collegiate athletes preferred to source athletic trainers (39%), and strength and conditioning coaches (23%) for nutrition information more than dietitians (14%). This may suggest that even with increased availability of dietitians, integration and collaboration between qualified nutrition professionals, strength and conditioning coaches, and athletic trainers may be required to refer athletes to the dietician and thereby provide greater opportunities for athletes to interact with nutrition support services. Such an integrated approach is an important consideration as athletic training staff are noted to be key influencers on nutrition behavior and decisions, particularly in younger athletes [33, 34].

In the current study, the athletes’ knowledge of DRI was particularly lacking with majority of respondents incorrectly identifying recommended DRI values for carbohydrate, fat and protein, with mean correct scores of 26, 26 and 30% respectively. The high percentage of ‘incorrect’ and ‘unsure’ responses clearly demonstrates that regional youth athletes display a lack of DRI nutrition knowledge. Interestingly, athletes displayed higher knowledge in the subcategory of weight loss compared to weight gain, with more correct (59% vs. 42%) and fewer unsure (28% vs. 32%) responses, respectively. This contrast may be reflective of increased exposure to health promotion campaigns and social media targeted at reducing obesity in the Australian population, as an inundation of information regarding weight loss is highlighted throughout all media outlets. This may lead to youth athletes questioning whether it is healthy and/or acceptable to increase their energy intake despite well-established guidelines that youth athletes require greater caloric intakes to support growth, development and physical activity [3, 4].

The supplementation subcategory displayed the second least ‘correct’ responses across all categories with greatest number of ‘unsure’ responses. This further supports the works of Little et al. [31] who highlight that youth athletes have poor understanding of concepts related to nutrition supplements. Specifically, related to protein supplementation, Petróczi and colleagues [35] reported 21–44% of young elite UK athletes (12–21 yrs) supplemented with whey protein. This is consistent with the current investigation wherein 31% of youth athletes reported protein supplement use. It should be noted that while there was a high “unsure” response rate to creatine and HMB (63 and 70%, respectively), these are quite technical terms and potentially unknown to youth athletes. More than reflecting their knowledge and therefore the health hazard it might also convey the athletes are unfamiliar with these kinds of supplements. Collectively, these data indicate that not only do youth athletes use dietary supplements, but perhaps more importantly, they lack knowledge pertaining to the efficacy and risks of protein supplements. Self-managed supplementation strategies employed by youth athletes may partially explain the observed lack of congruence between reasons and supplement used [35].

As with all research there are potential limitations that should be acknowledged. Firstly, difference in the male and female sample size are apparent and while significant difference observed in the nutrition category may be due to the unequal sample sizes the statistical analysis and framework used in the manuscript is consistent with publications in the area. Secondly, the NKQ used was based on a questionnaire previously determined to have construct validity [24], however some questions underwent minor word changes to account for the specific population studied, that of regional academy youth athletes. While the overall questions remained the same, some questions were rephrased to be in the second person tense. As highlighted by Abbey and colleagues [36], acknowledging that no questionnaire is completely robust, the chosen NKQ was deemed the easiest to assess nutrition knowledge in regional academy youth athletes.

## Conclusion

In agreement with previous research [13, 14], it was evident that non-residential Academy of Sport youth athletes from regional and rural areas displayed several misconceptions regarding general nutrition. Specifically, DRI and supplementation have been identified as key areas requiring targeted education. Secondly, with the tendency for athletes to source nutrition information from coaches/trainers, general nutrition education for coaching staff is highly recommended as this may offer a flow-on effect for enhancing youth athlete nutrition knowledge via coaches’ reinforcement of sound nutrition principles [16, 20, 21, 37]. This is an important consideration given that few youth athletes seek nutrition information from appropriately qualified nutrition professional, especially in regional Academy of Sport programs that may lack such resources. A better understanding of nutrition knowledge in youth athletes will allow nutrition education interventions to target areas in need of improvement. These data provide further support to current recommendations that athletes [31] and coaches [16, 23] would benefit from nutrition education integrated into Academy of Sport programs conducted by qualified nutrition educators, especially for young male athletes.

## Data Availability

Availability of dataset can be given upon reasonable request to the first and/or corresponding author.

## Abbreviations

DRI: Dietary reference intakes
HMB: hydroxy-methyl butyrate
NKQ: Nutrition knowledge questionnaire
WRAS: Western Region Academy of Sport
